# Urban land for a growing city at the banks of a moving river: Vienna's spread into the Danube island Unterer Werd from the late 17th to the beginning of the 20th century

**DOI:** 10.1007/s12685-013-0078-y

**Published:** 2013-07-03

**Authors:** Gertrud Haidvogl, Marianna Guthyne-Horvath, Sylvia Gierlinger, Severin Hohensinner, Christoph Sonnlechner

**Affiliations:** 1 Department for Water, Atmosphere, Environment, Institute of Hydrobiology and Aquatic Ecosystem Management, University of Natural Resources and Life Sciences Vienna, Max Emanuelstraße 17, 1180 Vienna, Austria; 2 IFF- Faculty for Interdisciplinary Studies, Institute of Social Ecology, Alpen Adria Universität Klagenfurt-Graz-Wien, Schottenfeldgasse 29, 1070 Vienna, Austria; 3 City Archives of Vienna, Guglgasse 14, 1110 Vienna, Austria

**Keywords:** Vienna, Danube, Danube floodplains, Land use history, Urban development, Flood protection

## Abstract

In the relation between urban development and the Viennese Danube different periods can be identified from the late 17th to the early 20th century. These periods were strongly intertwined with both the history of the river and the history of the city. Urban expansion into the floodplains is demonstrated in this paper by investigating the island Unterer Werd, next to the city centre. In the late 17th century the fluvial dynamic still hampered urban development on the island. First measures to stabilise the river banks and to protect buildings from floods were taken soon thereafter, but the majority of practices aimed at mitigating the risks and impacts of the frequent floods: inundation was a part of the arrangement and the main target was to minimise the potential impacts. This practice also prevailed after the 1830s, when urban expansion began to move into the north and northwest of the island and the Danube floodplains were considered an important land resource for the growing city. In connection with new technologies and available means to channelise the river, the relationship between Vienna and the Danube changed fundamentally. Urban development in the riverine landscape gained new momentum. This process was initiated before the Great Danube Regulation from 1870 to 1875 was completed, the rate of growth accelerated after 1875. The last decades of the 19th century mark a turning point in the urban development of Vienna, with expanding urban areas becoming dependent upon a well functioning and maintained flood protection system.

## Introduction

Today approximately 35 km^2^ or 24 % of Vienna′s urban area is situated in the former Danube floodplains. Urban development and the Danube waterscape have been linked for centuries. However, the large-scale integration of the floodplains was initiated only after the construction of a complex and labour-intensive flood protection system in the 1870s. Flood security was improved by several further measures which were implemented in particular in the 1970s, when a 21 km long flood bypass was excavated parallel to the main Danube channel. From the mid-19th century onward, urban development was a main driver for major changes in the riverine landscape. Today, immense economic value is inextricably linked to the former floodplains and continuous investment to maintain all the technical facilities required to prevent inundation is therefore indispensable.

The Great Danube Regulation fundamentally modified the relation between the river and the city. Nowadays the river—or to be more precise, the four different channels of the Viennese Danube—are perceived as stable areas where water and land are clearly delineated. Before channelisation, the fluvial system comprised constantly moving areas of water and land, which posed considerable challenges for the foundation of urban areas.

This article contributes to the growing field of environmental and urban history of rivers (see e.g. Mauch and Zeller 2008; Castonguay and Evenden 2012). It addresses the interaction between a large river and urban development before and during industrialisation. In contrast to topics such as the urban uses of rivers (e.g. Kelman 2006), river pollution (e.g. Jørgensen 2010) or studies on the effects of floods (e.g. Lübken 2012), the impact of the environment in general and rivers in particular on the urban fabric and vice versa has far less commonly been the focus of research (Reynard 2010; Castonguay and Dagenais 2011). Vienna is an interesting case because, in contrast to many other European metropolises, it developed along the upper section of a large river which shows strong alpine influence, i.e. rapid flow and high fluvial dynamic. As the capital of a huge empire it was the seat of the emperor, the location of various administrative and governmental institutions and a place for trade and commerce. Industrialisation and urbanisation triggered an enormous demographic growth in the 19th century. Urban authorities were confronted with new challenges including the provision of appropriate land resources for the population and for industry.

Our investigations, which build on the findings published by Haidvogl (2012), cover the period of roughly 200 years from the end of the 17th century to the beginning of the 20th century, when the Danube was converted from a hydromorphologically dynamic river to a tamed urban waterway and energy producer. Prior to channelisation, all the floodplains were exposed to the hydrological dynamic, i.e. they were regularly flooded, although not all zones were affected to the same degree of intensity. The morphological dynamics differed within the vast riverscape, creating places of varying suitability for settlements. Furthermore, the urban interest in colonising floodplains was not equal for all zones. The most attractive were areas opposite to the historical town that was situated on the western banks of the Danube system on a Pleistocene terrace. Any connection to the northern parts of the Habsburg Empire thus had first to cross the various river arms and islands. But local connections to the opposite river banks were also important because of the villages there supplying the town with grain.^1^

Scrutinising Viennese maps from the late 17th to the middle of the 19th century makes obvious that the Danube floodplains had been a clear limit for urban expansion for many centuries. There was only one location inside the floodplains that was already urbanised by the late Middle Ages. This was the *Unterer Werd* island opposite the historical city centre which forms the core of our small-scale study. The island was given the name “Leopoldstadt” after the banishment of the Jewish Ghetto located there from 1624–1670 and today comprises the Viennese districts Leopoldstadt and Brigittenau. During the period covered by our study, several phases of interaction between urban development and the riverine landscape can be distinguished, in particular when looking at the extent and location of urban area as material arrangements and practices of flood protection.

A question addressed in this paper concerns the relation between the Danube, the Danube floodplains and urban development. We investigated where urban settlements on the island were situated at the end of the 17th century and how this changed between then and the beginning of the 20th century. This analysis is based on digitised maps from 1704, 1773, 1829, 1849, 1875 and 1912. The maps have different origins and purposes but all show settlements in sufficient detail and spatial accuracy. Methodological implications of the use of historical maps for spatial analyses are dealt with in detail in Hohensinner et al. (2013b, in this issue) but also in other studies such as Stein (2007) or Forget and Bravard (2011). We also describe briefly the economic and social functions of the island by analysing important economic and administrative activities and—depending on the availability of information—the social and professional background of residents and house owners. Further, by investigating documented flood protection measures we trace the development of practices to cope with the risk of flooding.The last section of the paper depicts the changing importance and role of the Danube floodplains for the urbanisation of Vienna between the late 17th century and the early 20th century, comparing the development in Leopoldstadt with that in other Viennese districts and by summarising urban planning activities and projects in the second half of the 19th century.

## Hydromorphological dynamic and urban land on the island until the late 17th century

The island as it appears in maps from the late 17th century was formed due to fluvial activities of the Viennese Danube between 1632 and 1663. Maps from around 1529 and 1570 and the reconstructions of the Viennese Danube landscape carried out by Hohensinner et al. (2013a, in this issue) show, in addition to the island *Unterer Werd*, several other small islands in the *Donaukanal* in front of as well as up- and downstream of the urban centre^2^. These small islands had disappeared by 1632 in the wake of large-scale morphological activities of the Danube resulting in the north-east shift of the main river arm away from the central town (Sonnlechner et al. 2013, in this issue). But in the first decades of the 17th century, the *Tabor arm* still divided the *Untere Werd* from a larger northern island that was to become the district Brigittenau in the 19th century. In the map by Priami from 1663, the *Tabor arm* had likewise disappeared except for a small remaining water body, due to processes of siltation.^3^

When the first buildings were erected in the *Untere Werd* in the early 14th century, it was thus just one of several Danube islands in close proximity to the city centre – even though it was one of the largest. It can be assumed that a key reason for the early urban colonisation was that, in contrast to many other islands, *Unterer Werd* remained fairly stable at least in its core area directly bordering the urban centre. The area of the island in 1529 comprised approx. 220 ha. By 1700, maps indicate an increase to 1,070 ha and by 1849 a further growth to approx. 1,900 ha; since 1875 the area of the island has amounted to 2,015 ha. The influence of human activities, primarily the Great Danube Regulation, on the present extent will be described below.

From mid-16th century maps and real estate registers, we know that houses already existed along the left banks of the *Donaukanal* in the late Middle Ages (shown e.g. in the map of Bonifatius Wolmuet 1547.^4^ See Steiner (no date, n.d.) for an investigation of real estate registers). However, maps produced before the second half of the 17th century are not accurate in terms of spatial location or dimension and it is difficult to evaluate whether the locations of the first buildings along the banks of the Danube channel changed because of erosion or siltation. Descriptions of real estate registers are likewise not precise enough to identify the exact position of the oldest buildings. Considering that in the first centuries of its existence the settlement was on several occasions the scene of sieges and battles (e.g. Matthias Corvinus in the 1470s and 1480s, first (1529) and second (1683) siege by the Ottomans, and in 1619 in the course of the Thirty Years’ War), during which most of the buildings were destroyed, it is not unlikely that buildings were re-erected at slightly different locations. Fires, as well as regular small and medium and some devastating floods, documented in descriptions of *Unterer Werd* and Leopoldstadt respectively may have also caused the re-erection of buildings (see Bergenstamm 1812; Weschel 1824). Hence, there are many reasons why buildings could have been demolished and it is perhaps not surprising that measures taken against frequent erosion of buildings through the Danube are not mentioned in late medieval legal regulation of *Unterer Werd* (Weistum Unterer Werd 1460 in Winter 1886).

**Fig. 1:**
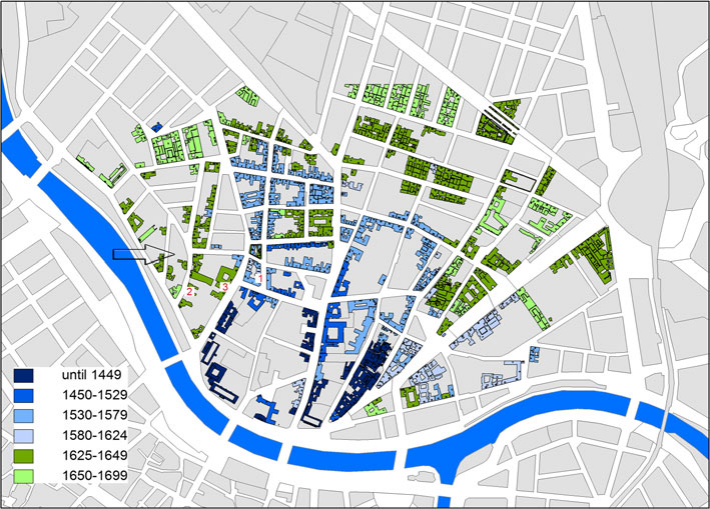
Buildings in *Unterer Werd* in 1699 projected onto the present day cadastral map. The different phases of urban development are indicated by* different colours*. Some buildings to which the text refers are marked (*1* Sperlgasse 3 and 5,* 2* Große Schiffgasse 4-6,* 3* Hollandstraße 9,* arrow* area of former Paderinsel, Floßgasse, Hollandstraße)

The interaction of urban development and fluvial dynamic along the banks of the *Donaukanal* up- and downstream of the medieval core area can nevertheless be traced from the mid-16th century onwards. Real estate registers from the 16th and 17th centuries (Rotter and Schmieger 1926; Schwarz 1909; Steiner n.d.) reveal the Danube dynamics as a decisive driver of urban development on the island. To take but a few examples from the historical evidence: For 1582 we read about a partly eroded parcel of land which had to be re-mapped before building a house (Fig. 1: Große Sperlgasse 3). Next to this place, an existing wooden building (*Waschhütte*) was recorded in 1568 as having been eroded by the Danube and only replaced in 1632 (Fig. 1: Sperlgasse 5). In 1631, two houses were built on former floodplains (Fig. 1: Große Schiffgasse 4–6, Hollandstraße 9). The name of this area refers indeed to an island - *Paderinsel*. In a graphic representation of the Viennese Danube landscape from 1570 (Hohensinner et al. 2013a, in this issue), this island can probably be identified as the one closest to the city centre. Most references to the colonisation of this former *Paderinsel*, by then termed a floodplain, date from the 1630s. The real estate registers also refer to buildings on former ditches or small pools, which had to be filled up before any construction works, as well as to small brooks and channels that still existed on the island at least in the 16th century. Notably, the location of the former Danube channel river banks and the Paderinsel can still be seen in the present structure of streets (see Fig. 1).

Figure 1 shows the temporal evolution of urbanisation in *Unterer Werd*, based on the investigation of real estate registers by Steiner (n.d.). Apart from intensified building activity in the 1550s and 1560s (see Sonnlechner et al. 2013, in this issue), a second key phase can be identified in the three decades after 1624, when a part of Unterer Werd was declared as the Jewish Ghetto. People who had been living in this area prior to the establishment of the Ghetto were offered new land parcels and houses outside of the Ghetto area by the Burghers’ Hospital.

## How an island becomes part of a metropolis: urban development and the river’s hydromorphology, 1704–1912

At the beginning of the 18th century, the populated area on the island—by then already known as Leopoldstadt—was considered to be a part of Vienna. Although legally Leopoldstadt did not belong to Vienna until 1850, it was integrated into the defence wall (*Linienwall*) built in 1704, which served also as an administrative and tax border between the city and the suburbs outside. Despite this integration via a military construction, the island lost its particular significance for defence interests in the 18th century and thereafter (see Sonnlechner et al. 2013, in this issue). Urban growth was increasingly organised by the city council, but the imperial court and aristocratic landlords also concentrated administrative buildings here for navigation and water engineering together with summer palaces, large parks and hunting areas.

When investigating the spatial evolution of urban land as arrangements and practices to cope with the dynamics of the Danube, three main phases can be determined in the period between 1704 and 1912:

1. From 1704 to 1837, urban development continued to concentrate on the older settled areas of Leopoldstadt. Flood defence was based mainly on practices to mitigate flood damages but in some places also on the erection of embankments and local flood protection dikes.
2. Between 1837 and 1875, we can observe a large-scale expansion of the urban area over the entire island on previously largely uninhabited places, but the flood protection practices still relied on the old system. During this time, the Danube floodplains gained a new role as an important land resource for the rapidly growing town. But one problem remained that still constrained urban development in the northern half of the island (Brigittenau): the groundwater table of the morphologically young floodplain terrain was comparatively high. Consequently, construction of roads and buildings called for extensive landfills that made such investments expensive.^5^
3. After the completion of the Great Danube Regulation in 1875, the interaction between the Danube riverscape and urban areas changed fundamentally due to the permanent fixation of the river bed and the separation of aquatic and terrestrial zones now free of flooding. Moreover, the groundwater table was also significantly lowered, which facilitated economic urban development (Kaiser 1966).^6^ In the following chapter sub-sections, these three phases will be described in detail.

### 1704–1837: Compression in and around the old urban area

**Fig. 2:**
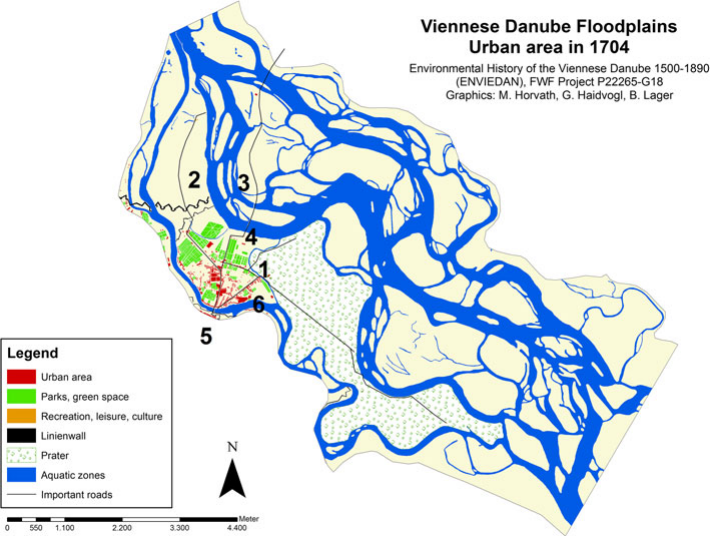
Urban area on the island Leopoldstadt and in the adjacent Danube floodplains in 1704. * 1* Fugbach,* 2* Fahnenstangenwasser,* 3* Kaiserwasser,* 4* Tabor,* 5* Schlagbrücke,* 6* present Franzensbrückenstraße

The outer limits of the urban area changed only slightly in the approximately 130 years between 1704 and 1837. When the first Viennese military barracks was constructed in 1723 (*Reiterkaserne*), it established the northern boundary of urban area for the century that followed. The southern limit expanded slightly between 1770 and 1837 to the area of the *Franzensbrückenstraße* (see Fig. 2). To the north, the Danube arm *Fahnenstangenwasser*, which was still large at that time, marked a barrier for buildings north of Augarten park and the Tabor toll station. Thus the main process in terms of urban development during this period was one of compression.

Beside the Danube river itself, we find that municipal authorities, regional and central institutions, together with burghers and aristocratic and ecclesiastical landlords and owners were important actors in either fostering or limiting urban development. In the 18th century, the municipality was already the biggest landlord in Leopoldstadt. In the northern part of the island outside the *Linienwall*, the Klosterneuburg monastery retained its manorial rights and property with few exceptions until the 1840s. The monastery made no systematic effort to establish urban areas on the island until 1837, when it started to sell land to real estate speculators. A special legal status was accorded to Augarten and Prater as imperial properties, whereby Augarten was enlarged considerably in the 17th and 18th centuries (Historischer Atlas Wien 1984 and 1987, maps nrs. 4.3.2. and 4.3.3). The Burghers’ Hospital lost most of its manorial rights in Leopoldstadt during the 18th century, retaining only the floodplain forests along the *Kaiserwasser* Danube arm. By 1825, small enclaves also belonged to aristocratic families and burghers.

Maps from this period depict dikes and embankments and show thus the efforts made to stabilise the banks of the Donaukanal and Fahnenstangenwasser. Historical documents refer to smaller floods occurring almost every year and also to severe floods e.g. in 1744 or annually between 1784 and 1787 (by then the biggest flood since 1501), as well as in 1813 (Weschel 1824; Kaiser 1966; Die Leopoldstadt 1937; Pasetti 1859). In 1744 the island remained inundated for one week and the inhabitants of Leopoldstadt had to be supplied with food from boats. During ice jams floods as e.g. in 1768 it could have happened that it was too dangerous to enter the island via boats because of floating ice sheets. People locked in their houses had to wait for hours or even days for rescue and help (Weschel 1824).

De Luca (1787) describes how the flooding risk depended on the topography of the island. The monastery of the Carmelites was the highest situated building, some parts of which were said to have never been reached by a flood. Other buildings in the vicinity and the hospital of the Brothers Hospitallers of St. John of God (*Spital der* *Barmherzigen Brüder*) were also rarely flooded due to their higher location. Both buildings were, for instance, not inundated during the large flood occurring in March 1744, when bridges and buildings were destroyed, and ships, rafts and fuel wood stored along the river banks were washed away (Wiener Diarium 1744). The practices for dealing with floods throughout this and the following period until the completion of the Great Danube Regulation in 1875 were to a large extent what river managers today term “passive” measures. Flooding was considered as a regular component of the arrangement. Practices were aimed at adapting the spatial location of buildings to the inundation risk, as mentioned above, and at mitigating the negative impacts. Different municipal authorities and in particular residents of the floodplains were involved in these practices. They were formulated in 1784 after a big flood and amended several times, significantly in 1799 (Bergenstamm 1812; Sartori 1830; Weschel 1824). One important measure involved removing ships and rafts from the *Donaukanal* each autumn when the shipping season came to an end because they would have increased the risk of ice jam floods by blocking the flow. The police service was allowed to take all barges and install them in those streets with high flood risk. In less risky areas, wood had to be prepared to install boardwalks in case of floods and construction wood to brace houses had to be made available. In case of floods, the Water Engineering Agency (*Wasserbauamt)* was responsible for closing the bridges to heavy vehicles and pedestrians. Ships to cut the ice on the Danube arms had to be ready as well as ships for crossing the *Donaukanal* to compensate for destroyed bridges. Bakers, butchers and other commercial food traders on the island were obliged to stock enough food for at least 14 days. The municipal council had to store flour in the monastery of Brothers Hospitallers and wood at the brewery. The monastery was also responsible for stocking drinking water in barrels. When floods were believed to be imminent, house owners were ordered to ensure older and ill people were moved to upper floors. Likewise, animals had to be brought to safe locations. During floods, soldiers were obliged to observe the water level and report houses at risk to collapse.

That these measures were carefully observed is well documented in Sartori′s description of the ice jam flood from the end of February to the beginning of March 1830 (Sartori 1830). What made this event so devastating was a too early all-clear given after the flood’s first peak and a decrease in the water level. The unexpected second wave hit many residents during the night without warning. More than 50,000 people were affected on both banks of the *Donaukanal* and 74 people died. Among the 49 victims of the Leopoldstadt community were 17 children. More than 430 people were rescued from their houses during the flood. The situation was in particular critical in the various charitable and public institutions, such as the hospitals or also the prison situated at Leopoldstadt, were residents and inmates had to be brought to higher floors. Also, some 100 horses and a similar number of cattle drowned. When the material damages were recorded after the water level decreased, 710 houses were found affected and partly or fully destroyed. The catastrophe initiated an enormous wave of helpfulness among wealthy Viennese citizens and especially indigent people got their damages recovered.

In order to show the evolution of urban development on the island between 1704-1837, we digitized information relating to three different points in time.

In 1704, the military engineer Leander Anguissola and the court’s mathematician Johann Jakob Marinoni drew a map of Vienna which for the first time also considered the urban fringe. While the depiction of the town was based on a wooden model by Suttinger from 1680, all other areas—thus also Leopoldstadt—were newly measured. The map was published in 1706.^7^ It shows housing blocks without differentiating single houses and despite the trigonometric measures, in comparison with the more detailed map of Arnold Steinhausen, which shows only a part of Leopoldstadt, the location of blocks of buildings does not always seem to have been precise.^8^

The urban area in 1704 amounted to approximately 20 ha. The total number of buildings is not available either from the map or from other sources. The only figures we have date from 1670, when the Jewish Ghetto was abolished. At that time, the Ghetto had 136 houses (Rotter and Schmieger 1926; Schwarz 1909; Weschel 1824 indicates 132) and Leopoldstadt outside of the Ghetto had 186 houses (Weschel 1824; Die Leopoldstadt 1937 reports 196), which makes a total of 322 buildings. Reliable population numbers are likewise not available for 1700. For the period around 1660, about 2,000 inhabitants are reported as living in the Jewish Ghetto (Die Leopoldstadt 1937). Taking into account that roughly 40 % of the buildings were located here, one could assume a total number of inhabitants of about 4,800 people yet given that roughly 16,000 inhabitants were reported as living there in 1777, this number was in fact probably higher (Weigl 2000).

In 1704, urban land concentrated on the left banks of the Danube channel, along the main road traversing the island and connecting the town to the north and along the main road to Prater (see Fig. 2). The limits of the settlement were determined by the location of still active (*Fahnenstangenwasser*) and aggrading Danube arms (*Taborarm*, *Fugbach*). Only a few local embankments and groins existed along the *Donaukanal* or at the right bank of *Fahnenstangenwasser* behind Augarten Park (Thiel 1904, 1906). There was still only one bridge crossing the *Donaukanal*. The *Schlagbrücke* was the oldest permanent Viennese bridge. It is mentioned for the first time in a decree of emperor Albrecht II from 1364 (Tomaschek 1877), which indicates the importance of the island as a connection route to the north.

By 1700, Leopoldstadt had become one of the larger Viennese suburbs (Eigner 1991; Eigner and Schneider 2005). Several aristocratic summer palaces together with their large parks dominated the appearance of Leopoldstadt in Anguissola’s and Marinoni’s map. Some of these were situated along the *Donaukanal* up- and downstream of *Schlagbrücke*, while others were spread over the whole urban area. Parts of the later Augarten Park were already the property of the imperial family since Emperor Matthias I had bought a land parcel in the former *Wolfsau* in 1614. In 1677, Emperor Leopold I bought the property of the aristocratic family Trautson, and his son and successor Joseph I ordered the first small castle to be built there. Due to its location within the Danube floodplains and the humidity, it was not used by the imperial family, at least in the first decades (Die Leopoldstadt 1937).

The former *Paderinsel* was completely connected to the main island. Most land parcels were transferred into construction areas during the period of the Jewish Ghetto, while existing water channels were actively filled by siltation (Weschel 1824). On the banks of the Danube channel, the map shows the main building of the Shipping Agency (*Oberst Schiffamt)*. This was transferred to the island in 1655 from the arsenal (Sonnlechner et al 2013, in this issue) within the city walls at initiative of the Court Treasure Chamber (*Hofkammer)* and the Court Council of War (*Hofkriegsrat)*, respectively, after the Danube shifted away from the town (Czeike 2004). The first building was enlarged in 1688 and existed at this location until 1843. Downstream from *Schlagbrücke* the imperial naval magazine (*Schiffstadel)* was situated on a favoured landing place for ships (*Am Gries*). The nearby meeting house of the fishermen’s guild was built in 1642 by the court fisher Simon Schmidt.

We have information from the real estate registers about the various professions of people owning houses in Leopoldstadt or paying taxes there. Although these figures do not allow us to gain a clear picture of the distribution of economic activities within Leopoldstadt, they do give an impression of the social composition of the resident population and its main economic focus upon water-based professions. The number of fishermen in the period 1648–1699 amounted to 106, and those involved in navigation to only 27, with both numbers having decreased compared to the first half of the 17th century. There were also 148 gardeners, who benefited from the good water supply close to the Danube arms (Steiner n.d.).

**Fig. 3:**
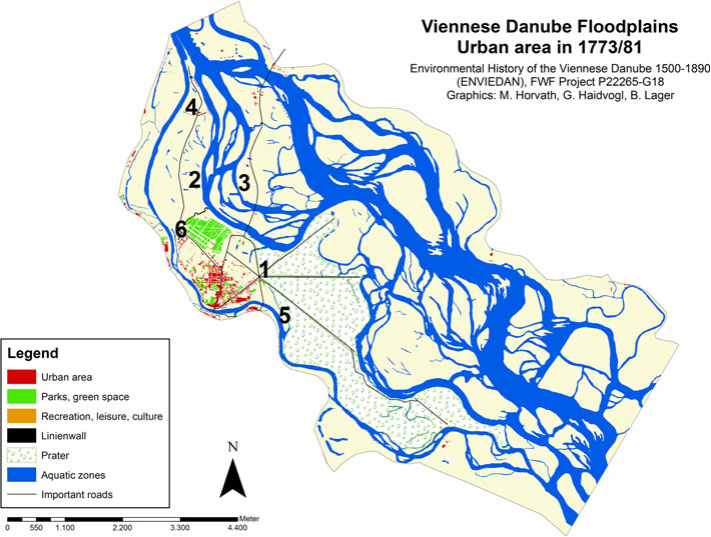
Urban area on the island Leopoldstadt and in the adjacent Danube floodplains in 1773/81.* 1* Fugbach,* 2* Fahnenstangenwasser,* 3* Kaiserwasser,* 4* Neuer Kanal,* 5* Schüttelbad,* 6* Reiterkaserne,* 7* Schlagbrücke

In 1767, the court mathematician Joseph Anton Nagel was commissioned by the Emperor to create a new map of Vienna and its suburbs within the Linienwall. The measures were completed in 1773, and the map was published in 1780/81.^9^ This was accompanied by a register of buildings, listing the names of the house owners.^10^ In this list we can find 398 houses in Leopoldstadt and a further 22 in the then independent municipality of Jägerzeile, which makes a total of 421 buildings. Another house register from 1779 (de Ponty 1779) recorded a total of 434 houses for both municipalities. The built up area was approximately 22 ha, which amounts to an increase of only 1.3 ha since 1704. The number of inhabitants was estimated as roughly 16,000 (Klein 2009; Weigl 2000). Compared to 1704, the limit of urban area had changed only slightly, meaning that the approximately 100 new buildings were built within the existing extent (see Fig. 2, 3).

Nagel’s map does not display dikes but in this respect we can determine the situation by investigating other maps and documents (e.g. Huber 1778)^11^. In addition to groins along *Fahnenstangenwasser* to protect Augarten Park from floods, several sections along the Danube channel were fixed with embankments, in particular up- and downstream of *Schlagbrücke*, around the area of the military barracks and especially along larger sections of the later district of Brigittenau, where the “new channel”, as it was named in Nagel’s map, had already been artificially created (upper part of the *Donaukanal*; Hohensinner et al. 2013a, in this issue). In 1770 there was still only one bridge connecting the island to the town. All further connections were made using ferries. The oxbows to the north of the island were smaller than they had been in 1704. The *Fugbach* was partly filled and only few years later, in 1789, was filled completely.

Among the major new buildings was the military barrack at the northwest end of the settlement, built in 1723. On the left bank of the Danube channel, the first swimming bath (*Schüttelbad*) was opened in 1717 at the initiative of the medical doctor Zehmayer. Water was extracted from the Danube channel and released back into it after use (Cerny 2010). The bath is not clearly indicated in Nagel’s map or in that of Huber, but it is shown in the detailed *Donaukanal* maps from Lantz made between 1802 and 1804.^12^

In 1770 most of the aristocratic parks along the Danube channel had already disappeared, some only very recently or around the time of the production of Nagel’s map. They were sold, and smaller houses erected shortly thereafter (Eigner 1991). Many small vegetable gardens still existed in around 1770, although a transformation into urban area can also be observed in respect of these. In contrast to Nagel’s map, that of Huber shows the large number of wells which had been constructed in these gardens to provide water supply.

From de Ponty’s (1779) list of house owners we can conclude that a diverse range of economic activities took place in Leopoldstadt where many administrative buildings related to navigation and river engineering still existed and individuals with water-related trades, e.g. fishermen and ship owners or gardeners were settled. According to Eigner (1991) and Lichtenberg (1977), a form of social segregation can be observed in the district, with a few remaining aristocratic summer palaces in the northern part, and hotels, restaurants and commercial trade along the main traffic routes. Day-labourers were in evidence but Leopoldstadt was still a place for wealthier burghers.

**Fig. 4:**
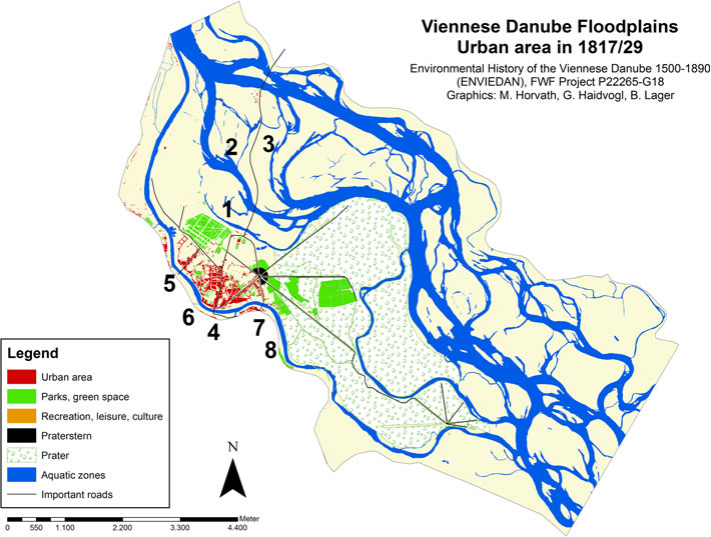
Urban area on the island Leopoldstadt and in the adjacent Danube floodplains in 1817/29.* 1* Fahnenstangenwasser,* 2* Kaiserwasser,* 3* Island Zwischenbrücken,* 4* Schlagbrücke (by then Ferdinandsbrücke),* 5* new street and bridge,* 6* Kaiser Karl Kettensteg,* 7* Franzensbrücke,* 8* Rasumowskybrücke

The cadastral survey of Vienna was completed in 1829, having started in 1818 (Opll 2004, 2nd edition). The map documents land use at the level of single parcels and is one of the most detailed and precise historical land use sources available for the entire present city (Fig. 4). For each cadastral municipality written documents and land use registers were additionally prepared. These are valuable sources for social and economic conditions as well as for agriculture and forestry. The accompanying documents for Leopoldstadt were completed in 1829 (forestry description and classification) and in 1830 (all other information).^13^

The number of buildings on the island had increased by 1829 to 709, while the number of inhabitants had reached 24,276. This enormous growth had taken place at a constant rate over the entire period from 1770: House registers for 1789 listed 515 buildings (Hofer 1789), rising to 589 buildings in 1812 (Fraißl 1812). The boundaries of urban area on the island had changed slightly. By 1829, the total urban area had increased to 39 ha, which was almost twice the area existing in 1770. The growth of settlements within the existing boundaries resulted in more densely populated zones especially in the north of Leopoldstadt and around Praterstern. These densely populated areas made up 21 ha compared to 8 ha in 1770, while less densely populated areas grew only from 2.6 to 6 ha. In 1829, a number of new buildings and technical facilities—e.g. for wood yards or bleaching—can be identified in previously uninhabited areas, either along the northern river banks of the island, in what would later become the district of Brigittenau, or close to the military barracks. Also on the large island known as *Zwischenbrücken* (meaning “between the bridges”) between the main arm of the Danube and *Kaiserwasser*, there were few houses recorded along the street traversing the Danube floodplains in a northerly direction.

In 1829, a complex system of dikes to prevent flooding existed along *Kaiserwasser* and *Fahnenstangenwasser*. The latter was already largely silted and partly filled and only a small body of water remained. The dikes were built between 1775 and 1793. Dikes along the *Donaukanal* were enlarged to keep the river bed within its course as well as to maintain and improve shipping access (see Hohensinner et al. 2013a, in this issue). When Obere Augartenstraße was developed as a causeway in around 1785, it had the function of a flood protection dike.

Residents, traders and the increasing number of temporary visitors to the leisure and entertainment places situated in Prater and Brigittenau required better facilities to access the island. Four new bridges had been built by 1829. From 1782 one of these bridges connected the city centre to the N*ew Street* to Augarten Park, which was opened to the public by Emperor Joseph II in 1775. Also from 1782, another bridge linked Leopoldstadt – and in particular Prater – to the Viennese district south of the city centre (the current third district, Landstraße). Further downstream, a third bridge was opened in 1792 (*Rasumovskybrücke*). From 1828 onwards, Salztorbrücke (then called *Kaiser Karl-Kettensteg*) located between Schlagbrücke and Augartenbrücke established another connection between the city centre and the island.

The river banks of the *Donaukanal* in Leopoldstadt had increased their importance for navigation. New landing places for wood shipments had been arranged in addition to the already existing landing place *Am Gries*, downstream of *Schlagbrücke*. Here a wood yard had existed since the 18th century, a grain magazine was built in 1804 and a second shipyard in 1812 (Die Leopoldstadt 1937). In the vicinity of this important landing place, the main office of the *Wasserbauamt* was built in 1780. In order to secure these buildings and facilities, the banks of the Danube channel were stabilised, an undertaking for which the *Wasserbauamt* itself was responsible.

Vegetable gardens retained their importance for the supply of food to the city of Vienna. The written documents of the cadastral survey offer an insight into how the gardens were distributed in terms of topography and soil.^14^ The more profitable category of garden was located in flat areas with moderate moisture. Here the soil was formed by sandy clay washed up by the Danube. A second category was situated on lower terrain, more often flooded than the first category and affected by a very high groundwater table. Economically, Leopoldstadt remained an important area for the city, although traditional professions and institutions involved in navigation, fisheries or vegetable gardens decreased during the first decades of the 19th century (Eigner 1991).

### 1837–1875: The transition phase–urban area on the island increases before the great Danube regulation

Between 1837 and 1875, aquatic areas on and around the island continued to decrease, although the Danube was still an important factor because of regular inundations. Two particularly devastating floods occurred in 1830 (see above) and in 1862. Both intensified ongoing discussions about the channelisation of the Danube and the implementation of a systematic flood protection system for Vienna. But it was only in 1870 that work on this large-scale project began. One reason for the continual delay concerned the lack of financial means (Thiel 1906). But there were also intense and controversial discussions among the engineers involved regarding the objectives of the regulation and the best approach to achieve them (Danube Regulation Commission (DRC) 1850; Danube Regulation Commission 1868). Flood protection was only one aim. Of equal or even higher importance was the improvement of the Danube as a shipping route and the erection of a permanent bridge to improve Vienna’s connection with regions to the north and northeast. In around 1850, a new aim was introduced into the discussions concerning regulation. As with many other European cities, Vienna saw enormous population growth in the first half of the 19th century and this was expected to intensify in the decades ahead (see Winiwarter et al. 2013, in this issue; Gierlinger et al 2013, in this issue). Thus urban land for residents as well as for commerce and trade was an urgently needed resource. The Danube channelization would support the reclamation of land because of reduced aquatic area and the establishment of terrestrial zones free of floods (DRC 1850; DRC 1868; Sax 1869).

Since the beginning of the 19th century, two channelization and flood protection principles were debated among the water engineers. In 1810, Josef von Schemerl, director of the Imperial Construction Council (*Hofbaurat*), developed a groundbreaking project based on novel technological principles. Schemerl was a well-educated person who favored and promoted the latest international developments in river engineering. He proposed to dig a new, straight main channel with only the Danube channel remaining as side arm for navigation into the city. Due to the support of count Wallis, president of the Court Treasure Chamber, Emperor Franz I approved the project in 1811 (Thiel 1906). Nevertheless, the implementation of Schemerl’s project failed not only because of the lack of money but also because of opposition, namely of count Herberstein, vice president of the Court Treasure Chamber. The latter succeeded in changing the Emperor’s mind who revoked his consent. Few years after Josef Osterlam, director of the Water Engineering Agency (*Wasserbauamt*), proposed another project, which represented the conservative approach: Osterlam’s project relied on the channelization of the existing main arm and the preservation of the sidearms. Oppositional engineering strategies and the involvement of different competing personalities as well as administrative and governmental institutions characterized the debate until the late 1860s when the final decision was taken based on the report of the second Danube Regulation Commission and four international experts who were invited to give their advice about the two main options (DRC 1868). The final decision for digging a new main channel was to a large extent owed to a new government installed in 1867 which favored this project (Thiel 1906).

The two options entailed different consequences for urban land in former Danube floodplains, the benefits and disadvantages of which were also debated among the members of the second Danube Regulation Commission (DRC 1868; Haidvogl 2012). Nevertheless, urban planners such as Heinrich Grave were not directly involved in these decisions but they commented on it in articles and maps.

We chose 1837 as one cornerstone for the delineation of this period because in that year Simon Eckstein, a real estate dealer, and Ignaz Mayer von Also-Rußbach, a wealthy wholesale trader, purchased 141 ha of land in the northern part of the island from the monastery of Klosterneuburg. Their original plans to establish shipping industry here with a harbour, industrial units and canals (Kaiser 1966, p 68) were not realized and they subsequently parcelled their land and leased it to private people. In 1858, the city of Vienna became the owner of this land. Most of the first residents were gardeners benefitting as in the southern part of the island from the flat terrain, the good water supply and the vicinity of the urban market for their produce.

Until the 1850s, the growth of urban land in the northern part of the island was not organised through spatial development projects. However, the municipal authorities and later the second Danube Regulation Commission ordered plans to initiate and support systematic urban development. The municipal council and architect Foerster completed a spatial development plan for the northern part of the island, by then already known as Brigittenau, in 1861.^15^ This was one of the first such initiatives, since for most other Viennese suburbs, the municipal authorities only acted as a planning institution after 1865 (Csendes and Opll 2006). During the Great Danube Regulation, spatial development plans for the entire city record expectations that new urban land linked with the Danube regulation would become available, as e.g. the map produced by Grave in 1874 or that published by the city of Vienna in 1870 (Grave 1874; City of Vienna 1870^16^). Brigittenau was dedicated as an area with cheap residential buildings in the centre and water-related commerce and trading close to the main arm of the Danube. The latter became the main navigation channel after the completion of the great Danube regulation in 1875. The easily available ground water and transport connections were considered advantageous for factories requiring large amounts of water (Kaiser 1966, p 144; GR Sitzungs-Protokoll 21.4.1863).^17^

**Fig. 5:**
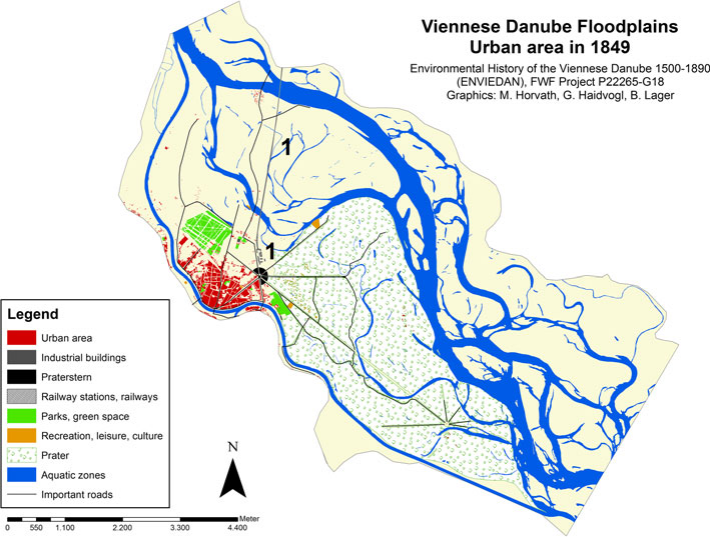
Urban area on the island Leopoldstadt and in the adjacent Danube floodplains in 1849.* 1* north railway link and station building

We have digitised the official “City map of Vienna” from 1849^18^ to analyse the evolution of urban land 25 years before the completion of the Danube regulation (see Fig. 5).

By 1849, the number of buildings on the island had increased to 874 and the number of inhabitants to almost 43,000 (Klein 2009; Weigl 2000). The total urban area comprised 57 ha, indicating an increase of almost 50% within 20 years. In 1850, Leopoldstadt was formally integrated into the city limits and together with Brigittenau formed the largest district of Vienna until the second major urban enlargement in 1890. The old central locations around *Taborstraße* and *Praterstraße* were still predominantly settled by middle class residents while in the newer quarters working class inhabitants began to dominate (Eigner 1991; Lichtenberg 1977).

The importance of Leopoldstadt as a Viennese transport hub was strengthened with the opening of the railway link to the north in 1837, the first railway line in the territory of the Austrian monarchy. As with any other site on the island, it was affected by regular flooding of the Danube. The bridges traversing the river arms were destroyed on several occasions and as in previous times, goods and passengers had to use ships and rafts instead.

### 1875–1912: Separating land and water–urban expansion on the entire island after the completion of the Great Danube regulation

Between 1870 and 1875 the Viennese Great Danube Regulation was accomplished by a French company which gained experience in such huge projects during the construction of the Suez Canal. Thousands of low-paid workers were hired, many of them from other areas of the Austrian Empire as e.g. from Bohemia, Slovakia, Hungary, Italy and Poland. They lived in temporary, wooden huts under bad hygienic conditions. Diseases were frequent and it is likely that the first cholera epidemic in Vienna had its origin in these barracks (Klusacek and Stimmer 1995).

The completion of the channelization made available new land while previously terrestrial areas, parts of the former island *Zwischenbrücken*, became aquatic. This process of transformation, which had prevailed and characterised the area for millennia, happened for the last time and not in the form of a natural process of erosion and sedimentation but as an enormous, concentrated investment of human labour and energy: now terrestrial and aquatic areas were determined and fixed for the future. Dikes prevented any flooding except in the defined inundation zone along the left bank of the main channel. The now dominant practice of flood protection required the continuous investment of energy and labour for maintenance. Surveys of the discharge capacity of the river bed and expert opinions soon demonstrated that the new river bed was not adequate for floods over a return period of 100 years (Waldvogel 1905). Thus discussions about flood protection continued and further measures were called for.

The newly established terrestrial areas along the new main Danube channel were owned by the second Danube Regulation Commission, which together with the municipal authorities propagated urban development and especially commerce and trading on the island. The practices of systematic urban planning in the new terrestrial zones are also mirrored in the establishment of the sewage system. Unlike in the older parts of the island, the construction of the sewage system in the new areas was projected and planned. Brigittenau was one of the first places in the city where the construction of an integrated and uniform sewage system was undertaken (see Kortz 1905). As in London, which formed the model for this undertaking, they built intercepting sewers, gathering the smaller sewers from the houses and streets and discharging the sewage at one spot. In the older parts of the island in Leopoldstadt, sewers also existed, but these did not meet the sanitary standards of the late 19th century. Many small sewers took the shortest route to the *Donaukanal*. When there was a high water level there, sewage was retained due to backwater. In the second half of the 19th century a great part of the sewage of the whole city was still discharged into the *Donaukanal* (see Gierlinger et al. 2013, in this issue). Fixing the water table and establishing large scale intercepting sewers were urgently required measures to protect the urban area in the former floodplains around the *Donaukanal* from polluted water (Kohl 1893; Berger 1894; WSTP Wiener Stadtphysikat 1864–1913). Between 1893 and 1894, the intercepting sewer along the *Donaukanal* on the left side was built, while the older facilities in Leopoldstadt were renewed (Kohl 1905). The system on the island became part of an integrated waterborne sewage system running through the entire city.

Two maps that were digitised indicate the growth of urban land after the Danube regulation was completed. One displays the situation in 1875 right at the end of the great Danube regulation, and the second shows the development by 1912, a period when Vienna reached its largest extent in terms of population numbers.

**Fig. 6:**
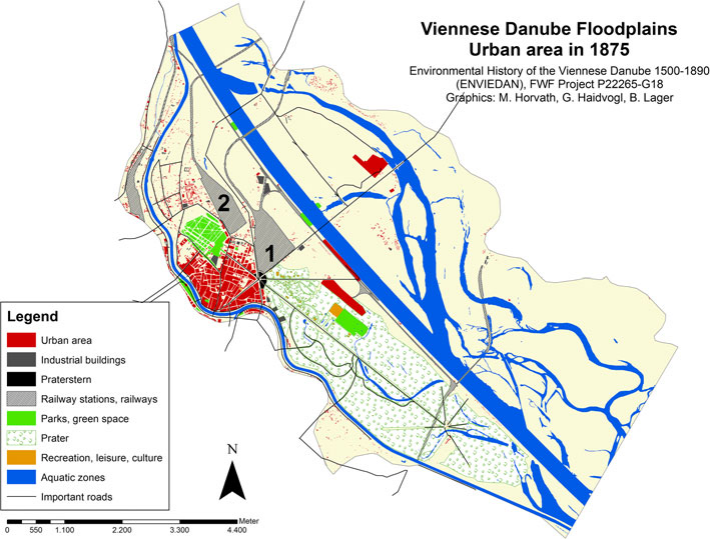
Urban area on the island Leopoldstadt and in the adjacent Danube floodplains in 1875. *1* enhanced north railway,* 2* northwest railway

The situation in 1875 is based on the third military survey from 1875 (Fig. 6).^19^ It was mapped using the new decimal scale of 1:25,000 by the official military mapping agency (*Militärgeographisches Institut*) and covered the entire Austro-Hungarian monarchy. The growth in the number of buildings, urban area and population reflected in the military map of 1875 and in statistical surveys of 1880 (Hickmann 1903, Statistisches Jahrbuch der Stadt Wien 1884) show the enormous impact of urbanisation on the island. The population had grown six-fold since 1850 and amounted in 1880 to almost 119,000. The number of buildings had doubled from approximately 870 to c. 1,650 between 1850 and 1870 and had risen to more than 2,000 by 1880. Urban area had also doubled between 1849 and 1875, when 100 ha of the island were urban land of which 80% was categorised as densely populated.

As Fig. 6 shows, most former river arms had disappeared by 1875. The remainder, the *Kaiserwasser*, were actively filled during the Danube Regulation with the material excavated from the new main river channel. Furthermore, the new terrestrial areas along the main channel were heightened using the excavated material. New connections to harbours, railway stations and railways, and new bridges were constructed. The north and the north-west rail links, built during the Great Danube Regulation, began to dominate the appearance of the island and disconnected the western, urban-oriented part of the island from the eastern part.

**Fig. 7:**
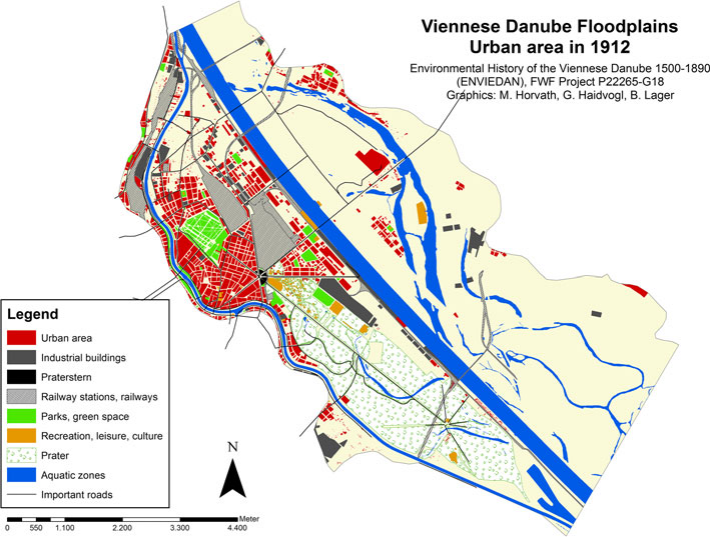
Urban area on the island Leopoldstadt and in the adjacent Danube floodplains in 1912

The final temporal point analysed in our investigation was digitised based on the municipal development plan from 1912.^20^ This official plan was based on a survey of different years and represents in fact the situation over several years. As Fig. 7 shows, large commercial and trading building blocks existed along the new main Danube channel. A railway (*Donauuferbahn*) along the main Danube channel, finished in 1880, connected the new harbours, landing places and urban railway lines. The shift of the transport facilities caused also a transfer of the main urban water front from the *Donaukanal* to the new main channel of the Danube on the eastern side of the island. Parallel to the Great Danube Regulation, the channelisation of the Donaukanal was completed. By 1912, it was already flowing between artificial flood protection levees.

Both the number of buildings and the number of inhabitants more than doubled again after 1880 (with 4,040 buildings, and c. 268,600 inhabitants, according to the Statistisches Jahrbuch der Stadt Wien 1913). Urban land had increased by 120 % since 1875 and in 1912 covered more than 221 ha. The increase is observable mainly to the north of the Prater amusement park as well as in Brigittenau, which became an independent Viennese district in 1900. Figures about specific water-related occupations established on the island are not available, but we do know, for example, that in 1910 10 out of 22 Viennese fishermen still lived in Leopoldstadt. The statistical survey from 1910 indicates that Brigittenau was a district mainly populated by workers and poor people: almost a third of the district’s inhabitants were employed as workers and almost 50 % of people living there were classified as “relatives without professional occupation.” In Leopoldstadt, the proportion of inhabitants employed as workers was only 20 % while relatives without professional occupation amounted to 40 % (Statistisches Jahrbuch der Stadt Wien 1913).

## Urban land in the Danube floodplains and the development of Vienna

**Fig. 8:**
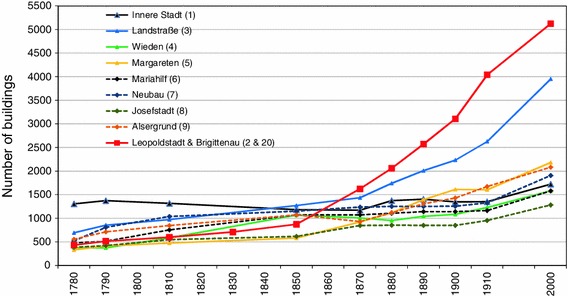
Number of buildings in the city centre of Vienna and the eight suburbs integrated within the city border in 1850 (data from de Ponty 1779, Hofer 1789, Fraißl von 1812, Franziscan Cadastral Survey 1817–1829, Hickmann 1903, Statistisches Jahrbuch der Stadt Wien 1913). The numbers of the districts are given in brackets

Despite the island being at least partially flooded almost every year up until the completion of the Great Danube Regulation, it had been an important Viennese suburb for centuries. Figure 8 shows the growth in the number of buildings in the city centre and the eight surrounding suburbs for the period 1780–1910. Figures for the year 2000 are given in addition, to show the spatial potential for further buildings until the present, with most districts now being completely populated. Leopoldstadt was an important urban area throughout this period but until the late 18th century, the development of Vienna was concentrated to the west and south of the centre (Eigner & Schneider 2005). In the 19th century, the role of the island for urban development also increased due to the large spatial potential it represented. By 1870, the number of houses in Leopoldstadt exceeded that in all other historic Viennese suburbs—a process which continued through the following four decades. The figures clearly confirm the expectations of the city in the land resources to be gained through the Danube regulation. Most new buildings were erected in Brigittenau. The population numbers there increased in the latter district from c. 3,600 in 1857 to c. 17,250 in 1880 and to more than 100,000 by 1910 (Kaiser 1966 p 272–275; Statistisches Jahrbuch der Stadt Wien 1913).

The colonisation of the island *Unterer Werd/Leopoldstadt* is the most striking example of the integration of the Viennese Danube floodplains into the urban area. As stated in the introduction, most other regularly flooded zones formed a clear limit to urban expansion until the 19th century, when some areas on other islands—namely *Zwischenbrücken*—and on the left river bank began to undergo development (Haidvogl 2012). This process continued in the second half of the 19th and in the 20th centuries. The large scale utilization of urban areas and the construction of infrastructure seemed especially in the first decade of the 20th century important when Vienna expected as capital of the huge Habsburg Empire a population growth of up to four million people by the 1940s (Mayer 1972). Nowadays, the floodplains are completely urbanised with the exception of Prater and Lobau, two former imperial hunting grounds which were subject to specific regulation preventing urban development following the collapse of the Austrian-Hungarian monarchy.

The urban development of Vienna is not special when considering its principle location along a large river. Most large cities were founded along or around water, which provided important functions such as shipping, hydropower production, food supply, wastewater discharge, sometimes drinking water supply or in recent decades also recreation (see e.g. Backouche 2000; Backouche 2008 for Paris or Porter 1998 for London, to name just two important European examples). What distinguishes Vienna and the Danube, however, is the intense hydromorphological dynamic of the river and the centuries-long struggle to integrate the floodplains into the urban area. A similar example is Lyon which was founded at the confluence of the Saone and Rhone. The first is a slow flowing lowland river but the Rhone exhibits also a large alpine influence and the challenges posed to urban development were comparable to Vienna and the Danube. Lyon evolved on the right banks of the Saone and between the two rivers, but the left banks of the Rhone were a clear limit of the town until the first decades of the 18th century. Only after 1730 this area became a focal point of urban planners and all urban projects of the 18th century were shaped by the Rhone (Reynard 2010). A further interesting case is Strasburg, which was established on a stable island of the lower Ill at the limits of the Rhine floodplains. Thus the citizens of Strasburg had access to the Rhine via a small and morphologically quite stable river but they could avoid the risk of the fluvial dynamic emerging from the main transport route. An opposite example is Paris, which developed on an island of the Seine. The city benefitted from the direct access to the water way but there was the disadvantage of regular flooding and the drainage of swamps was required (Roux 2009). Also other large European cities such as Berlin or London evolved either already in the Middle Ages or in Early Modern times on both banks of their urban rivers. As Paris these two cities are located on the lower sections of their rivers close to the sea which results in limited morphological dynamics.

## Synthesis

In this study, we investigated the changes undergone by the large island *Unterer Werd* opposite the historical city centre of Vienna as a socio-natural site. We looked in particular at urbanised zones as material arrangements expanding in this period from 20 ha to more than 220 ha. Until the late 1830s, this increase concentrated around the settlement that had been established in the Late Middle Ages. This area was the morphologically oldest part of the island, while the northern and southern parts were established only in the middle of the 17th century due to the fluvial dynamics of the Danube. This dynamic continued to threaten the stability of land throughout the 18th century. Dikes and embankments were already an important element of the arrangements on the island in the 18th century. However, until 1875 these were local structures and their construction only one component of a complex set of practices to fix terrestrial areas and to prevent floods on the island. The main practice was what river engineers nowadays term “passive” practice: flooding was a regular part of the arrangement and the main aim was to mitigate the damage and loss of life therefrom. Not only were different municipal authorities involved in preparing for an expected flood event but also all residents on the island. The Great Danube Regulation changed these practices fundamentally. The main objective from then on was to separate land and water permanently and to keep the former floodplains free of floods at all times. While different municipal and federal authorities are still involved in this practice of active flood protection, residents are no longer concerned and affected. Flood protection measures are no longer linked to an expected flood but to anticipatory, continuous maintenance and the improvement of dikes and artificial river channels as a permanent component of the Viennese Danube floodplains as a socio-natural site.

## Footnotes

1 See e.g. Franciscan Cadaster Aspern 1829; Franciscan Cadaster Groß Enzersdorf 1828. Niederösterreichisches Landesarchiv (NÖLA), Landesfürstliche und staatliche Verwaltung bis 1945, Schätzungsoperate Nr. 32 and Nr. 193.

2 For simplicity we use the term Donaukanal here. For the development of this Danube arm, which was the main navigation route until the great Danube regulation, and its different names through time, see Hohensinner et al. (2013a), in this issue.

3 “Abriß zu Wien zu Versicherung der Brükhen”, Giuseppe Priami 1663, Oesterreichisches Staatsarchiv (OeStA) KA K VII e 152.

4 “Die fürstliche Stat wien in Osterreich wie Sy in Irem umbschwaif oder zarg beflossn. aus recht Geometruscher waß im grundt nidergelgt und …”, Wien Museum, Sign. 31.021.

5 Wex 1876a, 1876b; “Plan der gegenwärtigen Brigittenauer Ansiedlungs-Gestaltung”, Niveauplan der Brigittenau 1847, Stiftsarchiv Klosterneuburg Sp. 163 u. 171.

6 Wex 1876a, 1876b.

7 L. Anguissola and J.J. Marinoni, “Accuratissima Viennae Austriae Ichnographica Delineatio”, 1706. WSLA, Kartographische Sammlung, At 41.

8 Iosepho Augusto ichnographiam hanc imperialis suae sedis Viennae Austriae … dedicat … Werner Arnold Steinhausen. Ao. MDCCX. WSLA, Kartographische Sammlung 3.2.1.1.P2.1.234 G.

9 Grundriß der Kayserlich-Königl.en Residenz-Stadt Wine, Ihrer Vorstädte und deren anstoßenden Orte. WSLA, Kartographische Sammlung 3.2.1.1.P2.1.5/1. Ex.

10 Conscription der k.k. Residenzstadt Wien and Conscription gesamter Vorstädte der k.k. Residenzstadt Wien, Vienna 1770-1781. Österreichische Nationalbibliothek, Handschriftensammlung, Codex 12963 and Codex 12964.

11 Scenographie oder Geometrisch Perspect. Abbildung der Kayl: Königl: Haubt: u: Residenz Stadt Wien in Oesterreich. aufgenommen 1769–1773. WSLA, Kartographische Sammlung 3.2.1.1.P2.1.260.

12 Karte des gesamten Donaukanals, 1802–1804, J. Lantz, J. Schöps & M. Lebzeltern. Archiv Donauatlas MA 45 without signature

13 Franziscan Cadaster, Leopoldstadt, NÖLA, Landesfürstliche und staatliche Verwaltung bis 1945, Schätzungsoperat Nr. 215.

14 Franciscan Cadaster Schätzungsoperat Leopoldstadt. NÖLA, Landesfürstliche und staatliche Verwaltung bis 1945, Schätzungsoperat Nr. 215.

15 Brigittenau: Regulierungsplan, 1861. WSLA, Kartographische Sammlung, 3.2.2.P10/2.106126.

16 Wien vor und nach der Donauregulierung, 1870. Wien Museum, I.N. 52968.

17 WSLA GR Sitz. Prot. 21.4.1863.

18 Haupt- und Residenzstadt Wien mit sämtlichen Vorstädten und der Einteilung in Gerichtsbezirken. WSLA Kartographische Sammlung 3.2.1.1.P1.263.

19 Dritte oder Franzisko-josephinische Landesaufnahme 1867–1887. BEV, Historische Landkarten.

20 Atlas zum "Häuserkataster der k.k. Reichshaupt- und Residenzstadt Wien"; WSLA, Kartographische Sammlung 3.2.1.1.P2.3.1701.
